# Human lung cancer neutrophils generate NETs with preserved anti-tumor cytotoxicity but impaired anti-migratory activity

**DOI:** 10.3389/fimmu.2025.1643609

**Published:** 2025-08-25

**Authors:** Nuha Alsharif, Mohamad Qaisi, Merav Shaul, Naomi Kaisar-Iluz, Dan Padawer, Osnath Bouhanna, Yael Volman, Zvi G. Fridlender

**Affiliations:** ^1^ Institute of Pulmonary Medicine, Hadassah Hebrew University Medical Center, Jerusalem, Israel; ^2^ Faculty of Medicine, Hebrew University, Jerusalem, Israel

**Keywords:** neutrophil extracellular traps (NETs), direct-cell killing, tumor microenvironment, lung cancer, neutrophils

## Abstract

Neutrophil extracellular traps (NETs) are DNA-protein structures released during a form of programmed neutrophil death known as NETosis. While NETs have been implicated in both tumor inhibition and promotion, their functional role in cancer remains ambiguous. In this study, we compared the NET-forming capacity and functional effects of NETs derived from lung cancer (LC) patients and healthy donors (H). Neutrophils were isolated from peripheral blood and stimulated *in vitro* with phorbol 12-myristate 13-acetate (PMA) to induce NETosis. Isolated NETs were quantified and assessed for their cytotoxicity against A549 lung cancer cells and their impact on cancer cell migration. Whereas LC neutrophils (LCN) were less cytotoxic to tumor cells than H neutrophils (HN), their NETs maintained similar tumoricidal capacity – 41.6% ± 25.3% (LCN) *vs*. 46.4% ± 14.5% (HN), *ns*. Interestingly, we noted a correlation between the amount of NETs and their cytotoxicity to tumor cells. This effect could not be recapitulated with purified genomic DNA, inducing only 3.99% of cytotoxicity to tumor cells, and confirming that intact NETs are required for the anti-tumor activity. LCN displayed an increased frequency of NETosis following PMA stimulation, yet produced significantly fewer NETs per cell – 1569 ± 306 ng (LCN) *vs*. 2619 ± 313 ng (HN); p = 0.025. Reactive oxygen species (ROS) production was elevated in LC neutrophils, indicating that the NETosis defect was not due to impaired oxidative burst. LCN had increased expression of immunosuppression (PDL-1) as well as exhaustion and aging markers CD62L and CD11b). Only NETs from HN inhibited the migration of A549 tumor cells, whereas those from LCN failed to suppress, and in some cases appeared to enhance, cell motility. Our data suggest that NETs in lung cancer retain anti-tumor cytotoxicity capabilities but lose their anti-migratory capacity, highlighting their dual role in tumor biology and potential as therapeutic targets.

## Introduction

Neutrophils constitute a significant portion of the immune infiltrate in various cancer types, including lung, breast, melanoma, renal carcinoma, and more (1–4). However, their role in cancer has long been a matter of controversy. Tumor-associated neutrophils (TANs) have been shown to possess anti-tumor properties, such as direct cytotoxicity toward tumor cells and inhibition of metastasis (5–8). On the other hand, numerous studies have demonstrated that TANs can support tumor progression by promoting the angiogenic switch, stimulating tumor cell motility, migration, and invasion, and modulating other immune cells as part of the “immunosuppressive switch” (9, 10). The importance of circulating neutrophils, both in their direct role in tumor progression and as a “mirror” to TANs - is also complex and often bidirectional (11). Subpopulations of circulating neutrophils in cancer can currently be distinguished mainly by their densities. One subtype, with normal ‘high’ density characteristics (NDN), exhibits primarily anti-tumor properties as previously described (8). In contrast, another subtype, Low-Density Neutrophils (LDNs), consists of a mixture of mature and immature subsets (12). While the mature NDN subtype presents an N1-like phenotype and can kill tumor cells, LDNs are less cytotoxic and display impaired functionality and immune-suppressive properties (12).

In the past decade, a novel and exciting phenomenon observed in neutrophils is their ability to release neutrophil extracellular traps (NETs). NETs consist of fibrous decondensed chromatin released from neutrophils, along with histones, myeloperoxidase (MPO), and various cytoplasmic proteins (e.g., neutrophil elastase, cathepsin G, and lactoferrin) (13). Initially characterized as an antimicrobial mechanism, trapping and killing bacteria, the release of NETs has also been documented in the context of cancer and other diseases (14). However, the precise importance of NETs production in cancer remains to be elucidated. NETosis was initially described as a form of neutrophil suicide, distinct from both necrosis and apoptosis, following chemical stimulation with phorbol 12-myristate 13-acetate (PMA) (15). While the cellular mechanisms mediating “suicidal-NETosis” are still under study, a second major type of NETosis, termed “vital” NETosis, has also been described. It is proposed to be activated by smaller pathogens or physiological stimuli, such as activated platelets (16).

In the context of cancer, emerging evidence shows pro-tumorigenic roles of NETs, through cell damage and regeneration, leading to increased inflammation, and promoting tumor progression and dissemination (17). NETs have been mainly hypothesized to play a role in metastases, particularly due to their strong adhesive properties, allowing neutrophils to adhere to circulating tumor cells and potentially promote their transfer to distant sites (e.g. pre-metastatic niches) (18–21). NETs have also been described in cancer to promote immunosuppression in the tumor microenvironment (22). Antitumor effects of NETs have also been described, mostly depending on tumor type (23). This has been described as mediated through the degradation of cytokines and chemokines, thereby reducing sterile inflammation (24).

NETs interact with cancer cells and components of the tumor microenvironment, facilitating key processes such as tumor cell migration and invasion. NETs can directly influence cancer cell behavior. While histones within NETs can induce cell death in nearby cells, they may also activate pro-survival pathways in some cancer cells, enabling them to leverage NET-associated inflammatory signals to resist apoptosis (25). Another important mechanism through which NETs contribute to cancer progression is via remodeling of the extracellular matrix (ECM). Proteolytic enzymes present in NETs—such as matrix metalloproteinases (MMPs), elastase, and cathepsins—degrade ECM components, thereby creating a microenvironment conducive to tumor invasion and metastasis (26, 27).

Although the role of NETs in promoting tumor development and mainly metastases, appears more evident, there seems to be a bidirectional effect of NETs on cancer cell and cancer progression (27), and the hypothesis that NETs in cancer promote tumor progression and metastasis, has not been conclusively demonstrated. It is also possible that the accompanied release of Reactive Oxygen Species (ROS) together with the trapping of cancer cells could exert a cytotoxic effect and inhibit the dissemination of cancer cells. The pro- *vs* anti-tumor function of this phenomenon remains to be better clarified. Moreover, direct evidence for NETosis occurring under physiological conditions (as opposed to PMA, LPS, or fMLP stimulation) is lacking, and a more detailed description of this phenomenon is still needed.

In the current study we studied the direct effects of NETs produced from circulating neutrophils on human tumor cells. We aimed to evaluate the direct effect of isolated NETs without additional effects of neutrophils or other immune cells. Our overarching working hypothesis is that the dual interplay between tumor cells and neutrophils promotes the release of NETs, which, in turn, affect bi-directionally tumor progression and metastasis.

## Materials and methods

### NDN/LDF isolation from human blood

The studies involving human participants were reviewed and approved by Hadassah Medical Center institutional review board (IRB). The patients/participants provided their written informed consent to participate in this study, agreeing to the use of blood samples for research purposes and publication. Peripheral blood was collected from healthy donors and lung cancer patients. All samples underwent processing within 30 minutes of collection, and neutrophils were isolated as previously described (28). In brief, peripheral blood was collected into EDTA or heparin blood collection tubes, diluted with an equal volume of dextran 500 (3% in saline solution), (Sigma-Aldrich, Saint Louis, MO), and incubated for 40 minutes at room temperature to allow red blood cell sedimentation. The upper layer was then gently loaded on top of a 1077 Lymphoprep solution (StemCell Technology, Vancouver, Canada) and centrifuged at 400×g for 30 minutes at room temperature with no brake. The normal-density fraction pellet, containing NDN, was further subjected to red blood cell lysis.

### NETosis induction and NETs purification

After isolating the NDN from human blood, the NDN pellet was resuspended in RPMI 1640 (Sigma-Aldrich) containing 0.5% FBS. Three million neutrophils were plated in each well of a white 12-well cell culture cluster with a flat bottom, lid, and sterile plates (Corning, Summersville, MA), using 250 µl of RPMI 1640 with 0.5% FBS. After placing the cells into the wells, they were incubated for 30 minutes at 37°C and 5% CO2 before starting the stimulation. To stimulate the neutrophils, 1 µl of 500 nM PMA (Sigma-Aldrich) was diluted in 4.9 ml of RPMI 1640 with 0.5% FBS (ratio of 1:5000). Next, 250 µl of the diluted PMA was transferred into each well, making the final volume per well 500 µl, with a final PMA ratio of 1:10,000. After 3 hours of stimulation at 37°C and 5% CO2, the media were collected from each well by gentle pipetting into separate Eppendorf tubes. The tubes were then centrifuged for 5 minutes at 400Xg, and the supernatant was transferred into new Eppendorf tubes. Pairs of tubes were balanced to have the same weight, and the samples were centrifuged for 15 minutes at 14,000Xg to pull down the NETs. After centrifugation, the supernatant was discarded, and the pellet was resuspended in 22 µl of sterile PBS (Sartorius, Göttingen, Germany). The samples were preserved at -80°C for approximately one month.

### Flow cytometry

The cells were preserved in FACS buffer (PBS buffer containing 2% FBS, 1 mM EDTA, and 0.1% NaN2). They were incubated for 45 minutes at 4°C with fluorescently labeled antibodies and kept in the dark. After staining, the cells were washed with FACS buffer, centrifuged for 5 minutes at 300×g at 4°C, resuspended, and filtered for FACS analysis. All cells were stained with Pacific Blue-conjugated anti-human CD66b (Clone: G1015; Biolegend) to mark neutrophils. The additional anti-human antibodies used included CD62L-(Clone: DREG-56; Biogems); PD-L1-APC (Clone: MIH1; Biogems) and CD11b-PE (Clone: QA20A58; Biolegend). Data were acquired using an LSR-Fortessa Analyzer, and plot analysis was performed using FlowJo version 10.

### Evaluation of neutrophils’ ROS production

Isolated NDN from healthy and lung cancer patients were resuspended in Hank’s balanced salt solution (HBSS) containing 2% FBS. After that 180,000 cells/well were plated onto a white 96 flat-bottom well plate (Greiner bio-one, Kremsmünster, Austria). Immediately before reading, a solution containing HRP reagent (40 U/mL; Sigma-Aldrich) + Luminol (0.5 mM; Sigma-Aldrich) was added to each well. Following the addition of HRP and luminol, PMA (Sigma-Aldrich) at a final concentration of 20nM for human was added to part of the wells to compare ROS production with and without neutrophils’ activation. The final volume of each well was 200 µL. The chemiluminescence was read using a Tecan Spark 10M plate reader for 45 minutes at 37°C.

### Direct cell-killing assay

A549-luciferase tumor cells were purchased from the ATCC. Twenty-five × 10^4 cells were plated in 100 μL RPMI-1640 (Sigma-Aldrich) with 0.5% FBS in each well of a white 96-flat-bottom well plate. Subsequently, human purified NETs with a concentration of 2000–3000 ng/µl, previously isolated as described from either healthy donors or lung cancer patients, were added to the tumor cell culture. Following incubation, tumor cell death was assessed by measuring luciferase activity, using the Luciferase Assay System (Promega, Madison, WI), following the manufacturer’s instructions. Luciferase activity was measured using a Tecan F200 microplate luminescence reader. Cytotoxicity was calculating by substracting the remaining cells from the overall cells initially plated (%cell death = [1 – (positive cells/total cells)] X100).

### Wound healing assay

Neutrophils were isolated from human blood as previously described, and 150 × 10^3 cells were seeded in each well. NETs from either healthy individuals or lung cancer patients were added to the experimental wells at a concentration of 2000–3000 ng/µl. After creating the scratch, the plate was inserted into the IncuCyte device, which contains an incubator at 37°C with 5% CO2. Each well was captured every 30 minutes for 48 hours.

### Confocal microscopy assay for quantifying human NETosis

Following isolation, NDN or LDN were seeded in RPMI containing 0.5% FBS (Life Technologies), on poly-L-lysine (Sigma) coated coverslips (Bar Naor). Cells were left to adhere on the surface for 30 min at 37°C in medium before adding PMA (50–500 nM) or DMSO (at the respective dilution control, Sigma-Aldrich). Following 2 to 4 hours of incubation, cells were fixed with 4% PFA (Santa Cruz Biotechnologies, Dallas TX) for 1h at room temperature and permeabilized with 0.5% Triton X-100 in blocking buffer (PBS containing 5% BSA, Sigma) for 30 minutes at room temperature. Human neutrophils were stained with anti–Citrullinated histone3 (Abcam, Cambridge, UK) and secondary anti-rabbit AlexaFluor647 (Abcam), and anti-CD66b-FITC (BioLegend, San Diego CA). To facilitate the detection of intracellular and extracellular nuclear material, coverslips were mounted with Fluoroshield Mounting Medium containing DAPI (Abcam). NET formation was visually determined as the percentage of neutrophils identified positive for a DAPI, and CitH3 signal. Data images were acquired using a Nikon Confocal A1R microscope and were analyzed using ImageJ software. The number of NETotic cells was determined by another person that was blinded to the specific conditions.

### Statistical analysis

Statistical analyses were conducted using GraphPad Prism software version 8. Comparisons between two groups were performed using a one- or two-tailed unpaired or paired t-test as appropriate. For comparisons involving more than two groups, we employed ANOVA with multiple comparisons and applied appropriate *post-hoc* corrections. Differences were considered significant when p < 0.05.

## Results

### Lung cancer neutrophils are less cytotoxic to tumor cells than healthy neutrophils, while their NETs maintain similar tumoricidal capacity

**Figure 1:**
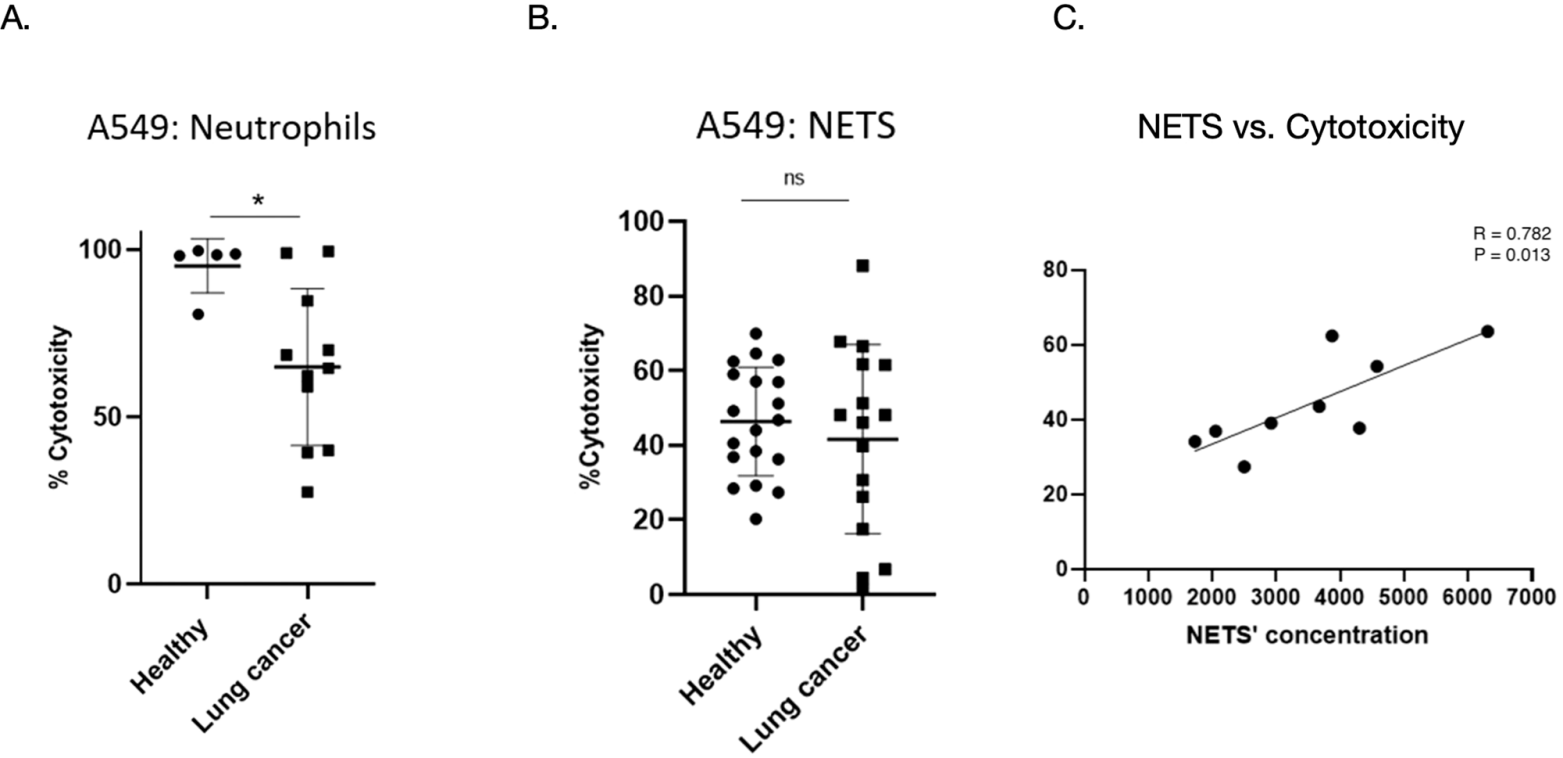
Cytotoxicity towards tumor cells of neutrophils and NETs from healthy donors and lung cancer patients. **(A)** Cytotoxicity of neutrophils from healthy donors (HN, n = 5) and lung cancer patients (LCN, n = 11) co-cultured with A549-Luc tumor cells in a 5:1 ratio. LC neutrophils had significantly reduced cytotoxicity (64.9% ± 23.5%) compared to healthy neutrophils (95.1% ± 8.1%); *p < 0.05. **(B)** Cytotoxicity of isolated NETs from HN (n=19) and LCN (n=16), normalized for concentration. No significant difference was observed (46.4% ± 14.5% *vs*. 41.6% ± 25.3%. *ns*, not significant. **(C)** Correlation between NET concentration and their cytotoxicity to tumor cells, showing a significant positive relationship (r = +0.782, p = 0.013). Data for A/B are shown as mean ± SD from independent donors.

To evaluate whether neutrophils from lung cancer (LC) patients differ in their ability to kill tumor cells, we co-cultured equal numbers of neutrophils from healthy donors (HN) and lung cancer patients (LCN) with A549-Luc lung cancer cells and assessed cytotoxicity via luciferase-based viability assays. As shown in **Figure 1A**, LC neutrophils exhibited significantly lower tumoricidal activity compared to healthy donor neutrophils (64.9% ± 23.5%) for LCN, *vs*. 95.1% ± 8.1% for HN; P = 0.0154 *p* < 0.05). This result suggests a functional impairment in the anti-tumor activity of neutrophils in the context of lung cancer.

We next assessed whether the cytotoxic potential of NETs differed between the two groups. Equal concentrations of isolated NETs from HN and LCN were incubated with A549-Luc cells in the absence of intact neutrophils. As shown in **Figure 1B**, both NET preparations induced comparable levels of cytotoxicity (46.4% ± 14.5% for HN *vs*. 41.6% ± 25.3% for LCN; *p* = 0.49), indicating that per unit NET, the tumoricidal function is preserved. To assess whether the quantity of NETs influences tumor cell death, we plotted NET concentration against cytotoxicity (**Figure 1C**). A significant positive correlation was observed (r = +0.782, p = 0.013), supporting a dose-dependent effect of NETs on tumor killing.

### Lung cancer neutrophils form fewer NETs per cell despite NETosis activation

**Figure 2:**
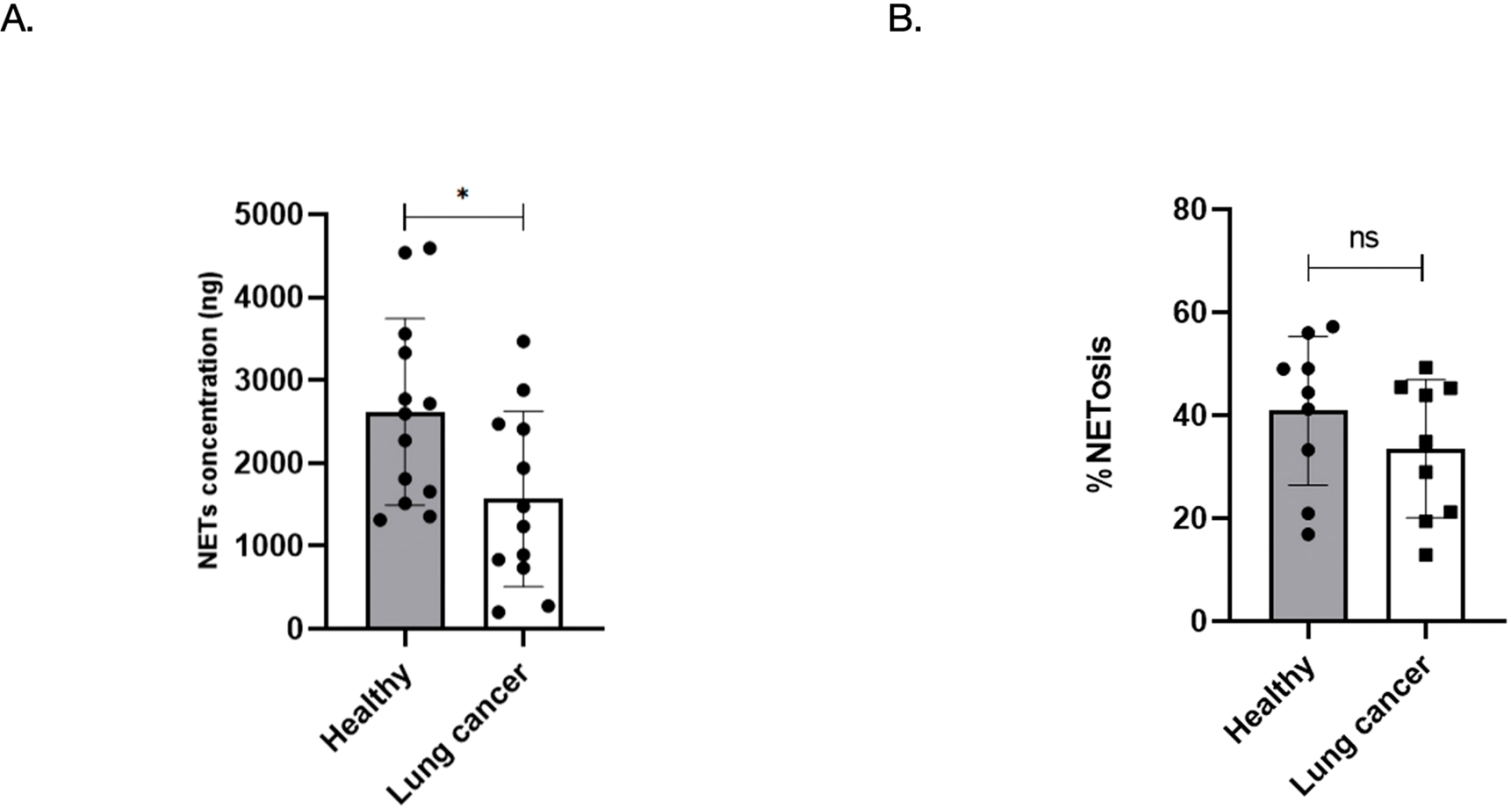
Neutrophils from lung cancer patients exhibit reduced NET production despite comparable NETosis frequency. **(A)** Quantification of NETs released by PMA-stimulated neutrophils from healthy donors (n = 10) and lung cancer patients (n = 13). Mean NET concentration was significantly lower in the lung cancer group (1569 ± 306 ng) compared to healthy donors (2619 ± 313 ng); *p < 0.05. **(B)** Confocal microscopy-based quantification of NETosis, defined as the percentage of neutrophils co-staining for citrullinated histone H3 and DAPI. No significant difference was observed between groups (mean 40.9% ± 14.5% for healthy *vs* 33.5% ± 13.4% for lung cancer; *ns*, not significant. Data represents mean ± SD. Each dot represents one donor.

To evaluate the NET-forming capacity of neutrophils from healthy individuals and lung cancer patients, we quantified NET production using two complementary approaches: biochemical measurement of NET-associated DNA and imaging-based quantification of NETotic cells. In the first assay, neutrophils were stimulated with PMA and total NET release was quantified based on extracellular DNA content. As shown in **Figure 2A**, neutrophils from lung cancer patients released significantly lower amounts of NETs compared to those from healthy donors (1569 ± 306 ng *vs*. 2619 ± 313 ng; *p* = 0.0251). This indicates a reduced overall NET output per a fixed amount of neutrophils in lung cancer patients.

To determine whether this reduction reflected a lower frequency of NETosis or merely decreased NET content per cell, we next assessed NETosis via confocal microscopy (**Figure 2B**). Neutrophils were stained for citrullinated histone H3 (CitH3) and DAPI, and the percentage of cells undergoing NETosis was quantified. Surprisingly, no significant difference was observed in the percentage of NETotic cells between the two groups (mean 40.9% ± 14.5% for healthy *vs* 33.5% ± 13.4% for lung cancer; P=0.2755), suggesting that the frequency of NETosis was comparable.

Together, these results indicate that while neutrophils from lung cancer patients undergo NETosis at similar frequencies as healthy controls, the total amount of NET material produced per cell is significantly reduced. This implies a qualitative impairment in NET production in the cancer setting, potentially reflecting altered chromatin decondensation or enzymatic activity downstream of NET induction. Although NETs from LC neutrophils retain functional activity, their reduced production in lung cancer patients could potentially diminish their cumulative effects.

### Lung cancer neutrophils generate higher ROS despite reduced NETosis

**Figure 3:**
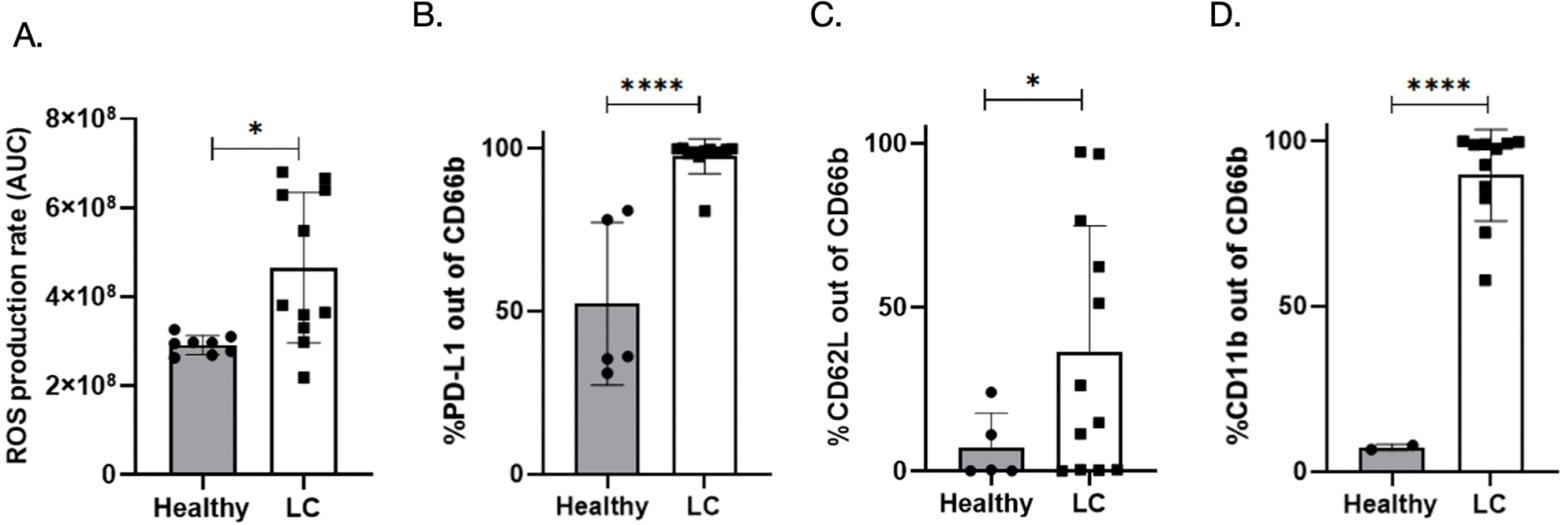
Modifications in LCN compared to HN – increased ROS generation and expression of exhaustion and immunosupression markers. **(A)** ROS production rate following PMA stimulation, expressed as area under the curve (AUC) of fluorescence intensity. Lung cancer (LC) neutrophils (n = 11) showed significantly higher ROS production (4.6 × 10^8^ ± 1.6 × 10^8^) compared to healthy donors’ neutrophils (n = 8), (2.9 × 10^8^ ± 2.1 × 10^8^); *p < 0.05. **(B)** Flow cytometric analysis of relevant surface markers on CD66b^+^ neutrophils. LC neutrophils exhibited significantly increased expression of PD-L1 compared to HN (97.5% ± 5.4% *vs*. 52.3% ± 24.9%). **(C)** CD62L (36.4 ± 38.4% *vs*. 7.1% ± 10.6%). **(D)** Elevation was seen in the expression of the degranulation/activation marker, CD11b (89.7% ± 13.8% *vs*. 7.3% ± 0.9%), *p* <0.0001. Data are presented as mean ± SD. (*p < 0.05, ****p < 0.0001).

Reactive oxygen species (ROS) are pivotal in driving NETosis by promoting chromatin decondensation and nuclear extrusion. To determine whether defective NET release observed in lung cancer neutrophils was associated with impaired ROS generation, we measured ROS production in response to PMA stimulation using a fluorescent ROS probe. As shown in **Figure 3A**, LC neutrophils produced significantly higher ROS levels than neutrophils from healthy donors (mean area under the curve [AUC]: 4.6 × 10^8^ ± 1.6 × 10^8^ in LCN *vs*. 2.9 × 10^8^ ± 2.1 × 10^8^ in HN, P= 0.0109, *p* < 0.05). Despite increased ROS output, NET production was lower in the LC group, suggesting that ROS generation per se is not limiting in this context. These findings indicate a disconnect between upstream ROS signaling and downstream NETosis execution in lung cancer neutrophils.

### Lung cancer neutrophils display markers of immunosuppression, exhaustion and cellular aging

To explore potential phenotypic correlates of neutrophil dysfunction, we analyzed surface expression of immune checkpoint and senescence-associated markers. Flow cytometric analysis revealed a dramatic increase in the expression of the immunosuppressive marker PD-L1 on CD66b^+^ neutrophils from lung cancer patients (97.5% ± 5.4%) compared to healthy controls (52.3% ± 24.9%**),** *p* < 0.0001 as shown in **Figure 3B** – left panel. LCN exhibited elevated expression of the resting/naïve marker, CD62L (36.4% ± 38.4% *vs*. 7.1% ± 10.6%**),** P=0.039 – **Figure 3C** – middle panel. Additionally, elevation was seen in the expression of the degranulation/activation marker, CD11b (89.7% ± 13.8% *vs*. 7.3% ± 0.9%**),** P= <0.0001, consistent with an aged and activated phenotype. as shown in **Figure 3D** – right panel. These markers are frequently associated with neutrophil exhaustion, chronic activation, and immunoregulatory dysfunction. Taken together, these data suggest that LCN exhibit phenotypic features of immunosenescence and exhaustion, which may underlie their impaired functional outputs, including reduced NETs.

### Purified genomic DNA fails to recapitulate NET-mediated cytotoxicity against tumor cells

**Figure 4:**
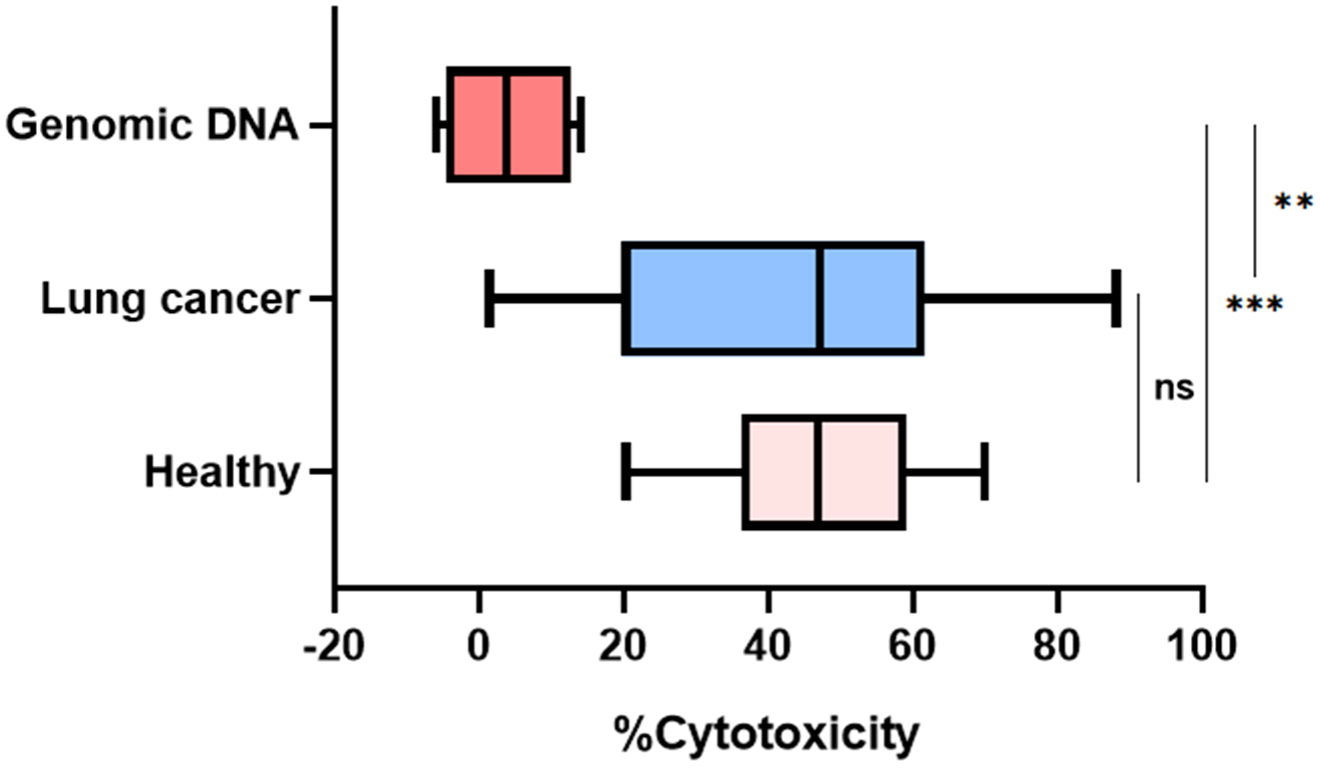
Cytotoxicity is induced by NETs but not by genomic DNA. Comparison of percentage of cytotoxicity in A549-Luc cells treated with purified NETs from HN or LCN, or with equivalent concentrations of purified genomic DNA. Genomic DNA (n=4) induced minimal cytotoxicity (mean 3.99%), significantly lower than both healthy NETs (n=19, 46.4% ± 14.5%) and lung cancer NETs (n=16, 41.6% ± 25.3%). There was no significant difference between NETs from healthy and lung cancer donors (*ns*), while both NET groups induced significantly higher cytotoxicity compared to genomic DNA. Data represents individual biological replicates; bars denote median and interquartile range. **p < 0.01, ***p < 0.001.

NETs are composed of DNA scaffolds complexed with histones and various neutrophil-derived antimicrobial proteins. To evaluate whether the observed NET-induced cytotoxicity is attributable to the DNA component alone, we incubated A549-Luc lung cancer cells with purified genomic DNA at concentrations equivalent to those found in NET preparations. As shown in **Figure 4**, treatment with naked DNA did not result in significant tumor cell death, in contrast to the marked cytotoxic effects elicited by intact NETs from both healthy donors and lung cancer patients. Quantitatively, the genomic DNA group displayed minimal cytotoxicity (mean 3.99%), whereas NET-treated groups showed substantially higher tumor cell death, mean 46.4% ± 14.5% for HN NETs and 41.6% ± 25.3%) for LCN NETs. Statistical analysis confirmed that cytotoxicity induced by genomic DNA was significantly lower than that induced by both healthy and cancer-derived NETs (p < 0.01 and p < 0.001, respectively), while there was no significant difference between the healthy and cancer NET groups (*ns*).

These findings demonstrate that the tumoricidal activity of NETs is not due to toxicity of naked DNA alone but likely mediated by the whole NETs complex or part of its associated proteins such as histones, neutrophil elastase, or myeloperoxidase. This control experiment underscores the specificity of NET-mediated cytotoxicity *vs*. other naked DNA.

### NETs from healthy donors inhibit lung cancer cell migration, whereas NETs from lung cancer patients do not

**Figure 5:**
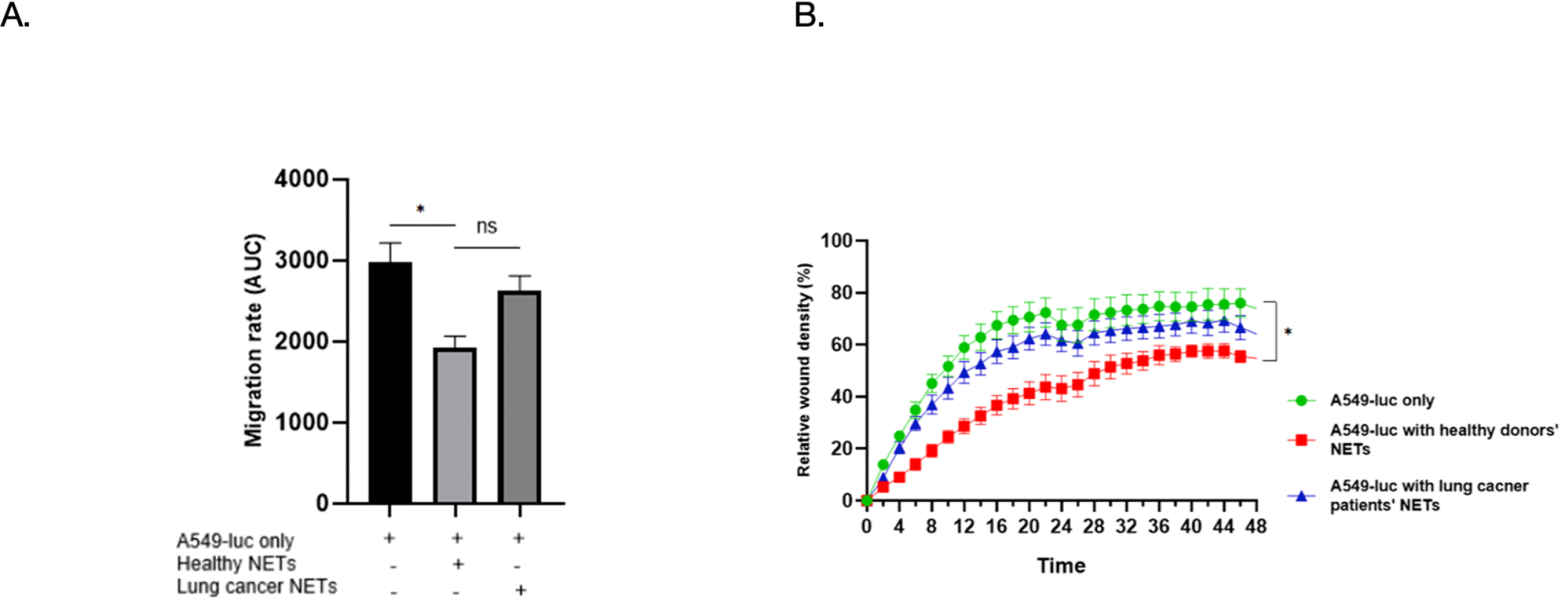
Differential impact of HN versus LCN NETs on A549 tumor cell migration. **(A)** Quantification of migration rate as area under the curve (AUC) over 48 hours. Healthy NETs (n=25) significantly reduced migration (AUC 1918 ± 41) compared to untreated controls (n=25) (AUC 2979 ± 81 AUC), while NETs from lung cancer patients (n=25) (AUC 2624 ± 65) showed no significant inhibition (*ns*). **(B)** A representative time-course of wound closure (relative wound density %) in A549-Luc cultures treated with NETs from healthy donors (red squares), lung cancer patients (blue triangles), or no NETs (green circles) is shown. NETs from healthy donors significantly suppressed migration compared to controls. *p < 0.05.

To assess whether NETs influence cancer cell motility, we conducted a wound-healing assay using A549-Luc lung adenocarcinoma cells in the presence or absence of isolated NETs. Scratch wounds were introduced into confluent monolayers, and cell migration was monitored over 48 hours. Quantification of migration rates, expressed as area under the curve (AUC), confirmed this observation (**Figure 5A**). A549-Luc cells without NETs (n=25) showed the highest migration rate (2979 ± 81 AUC), whereas treatment with healthy NETs (n=25) significantly reduced migration (1918 ± 41 AUC), p < 0.05). NETs from lung cancer patients (n=25) resulted in a modest reduction (2624 ± 65 AUC) that was not significantly different from the untreated control (*ns*).

As shown in a representative time-course example in **Figure 5B**, cells treated with NETs from HN exhibited significantly reduced wound closure compared to untreated controls, indicating that HN NETs impaired A549 cell migration. In contrast, NETs from LCN failed to suppress wound healing to the same extent, with A549 cells nearly closing the wound at a rate comparable to the untreated condition.

These data suggest that NETs from healthy individuals retain anti-migratory properties capable of restricting tumor cell movement, whereas NETs from lung cancer patients are functionally deficient in this regard. This highlights a qualitative difference in NET composition or activity between the two groups that may affect tumor progression and metastasis.

## Discussion

In the current study, we report for the first time a comprehensive evaluation of the direct multifaceted effects of isolated neutrophil extracellular traps (NETs) on human tumor cells *in vitro*. We first examined the capability of pure NETs derived from both healthy donors and lung cancer patients to directly induce cell-killing effects on cancer cells and found that different from their respective neutrophils, isolated NETs from both HN and LCN are cytotoxic to tumor cells in a similar level, with a direct correlation between the amount of NETs used and cytotoxicity to tumor cells. Furthermore, we observed that neutrophils from lung cancer patients secrete a lower concentration of NETs during *in vitro* stimulation compared to healthy controls. The production of ROS was higher in LCN compared to HN, suggesting that this is not the cause of the reduce production of NETs in circulating neutrophils from LC patients. However, LCN had more pronounced exhaustion and aging markers compared to HN, suggesting a potential reason for the reduction in NETs production in LC.

As mentioned, NETs have been implicated in cancer, but their exact role in cancer propagation remains unclear. It has been already shown by us and others that neutrophils can exert cytotoxic effects on cancer cells (29). Our results in the current study showed that neutrophils from LC have a lower cytotoxic impact on A549 cancer cells than healthy controls, consistent with previous findings (30). However, we show that isolated NETs can also induce direct cell-killing, suggesting that this mechanism is at least partly responsible of the cytotoxic effects of neutrophils on human tumor cells. Interestingly, previous studies have indicated that neutrophils from cancer patients, including lung cancer, produce lower levels of NETs compared to healthy controls under PMA *in vitro* stimulation, as we see in this study (31). Importantly, by comparing the effects of intact NETs with purified genomic DNA, we confirmed that the cytotoxic activity was specific to the complete NET structure and not due to naked DNA alone. Pure genomic DNA did not induce significant cancer cell death, supporting previous evidence that NETs’ cytotoxicity depends on the combined action of DNA scaffolding and attached granular proteins such as neutrophil elastase, myeloperoxidase, and histones (32, 33). Thus, “It’s the NETs and not the DNA”. Surprisingly, there was no difference between NETs isolated from HN *vs*. LCN in their cytotoxic effect on cancer cells.

ROS generation, a critical early event in PMA-induced NETosis and in NETosis in general (34), was unexpectedly higher in LCN compared to HN. Despite this, NET release per cell was significantly lower in lung cancer patients, indicating a decoupling between ROS production and effective NET formation. It is possible that defects occurring downstream of ROS generation, perhaps involving chromatin remodeling, nuclear envelope breakdown, or cytoskeletal rearrangements, are responsible for the reduction in NETs as previously suggested (35, 36). Moreover, our phenotypic analysis showed that neutrophils from lung cancer patients expressed elevated levels of exhaustion and aging markers, as well as the suppressor marker PD-L1. This finding aligns with prior studies linking PD-L1 expression to neutrophil dysfunction and immune suppression in cancer (37, 38). Aged neutrophils often display altered migratory behaviors, impaired antimicrobial responses, and reduced effector functions, all of which could contribute to the dysregulated NETosis observed in lung cancer (27, 39).

Recent studies have shown that isolated NETs can directly influence tumor cell behavior in pro-tumorigenic and anti-tumorigenic ways, and as mentioned above, the balance appears skewed toward supporting tumor progression (40–42). Our results actually suggest a new anti-tumorigenic effect by NETs, i.e. direct cytotoxicity. As much as we know this is the first study showing direct cytotoxic effects of NETs on tumor cells. This is described *in vitro*, and it is not possible to understand from our findings what would be the overall *in vivo* effect of NETs on tumor cells in the presence of the whole neutrophil and other cells of the immune system. Interestingly, the opposite effect has been suggested by Albrengues et al., who showed that Isolated NETs (purified from mouse lungs) directly stimulated proliferation of dormant breast and lung cancer cells (43). Yazdani et al. even showed that NETs augment growth of tumor cells (44). Our results seem to contradict these findings. The different methods and timing could be responsible for this difference, as well as the different species and cells studied. The correlation we found between the amount of NETs and the cytotoxicity of tumor cells (**Figure 1C**) supports the relevance of our findings. Further mechanistic studies are warranted. It is possible that NETs could be both cytotoxic to tumor cells on one hand and enhance their growth on the other side, and the total effect is the balance between these two.

Beyond direct cytotoxicity, we assessed the influence of isolated NETs on tumor cell migration. In this case we found a tumor-supporting effect of NETs, or at least an anti-tumorigenic effect of NETs, from healthy donors, inhibiting tumor cells’ motility that is lost in NETs from lung cancer patients. Our wound-healing assays revealed that NETs from HN significantly inhibited A549 cancer cells’ migration, whereas NETs from LCN failed to inhibit—and even appeared to enhance—cell migration. These opposing effects on motility could reflect differences in the molecular composition of NETs, likely shaped by the donor’s immune and tumor environment. Prior reports have suggested that tumor-conditioned neutrophils undergo phenotypic reprogramming that might affect their NET-associated protein cargo (45), potentially explaining the divergent effects on tumor cell behavior. This mechanism may add to a previous report by Yang et al. that NETs can promote adhesion of circulating tumor cells to endothelial surfaces and increase their migration (46).

Our findings in the current study underscore the dualistic nature of neutrophils in general, and NETs specifically, in cancer biology. While they maintain the potential to kill tumor cells, NETs—particularly those derived from the tumor-bearing host—may also contribute to cancer progression via lack of inhibition of tumor cell motility, that is seen with NETs from HN. This highlights the importance of dissecting the qualitative aspects of NETs, including their proteomic, genomic, and signaling profiles, in addition to their specific isolated functions. We are currently investigating the mechanisms of the difference in the amount and function of NETs between those isolated from healthy donor and lung cancer patients. Furthermore, we are studying the mechanisms by which NETs are capable of killing tumor cells directly. From a therapeutic perspective, understanding the contrasting functions of NETs may support the development of strategies that preserve or enhance their tumoricidal activity while inhibiting their pro-metastatic effects. Future studies should investigate whether pharmacological modulation of NETosis or selective targeting of NET components could shift the tumor microenvironment towards a more anti-tumoral state.

## Conclusion

Our data suggest that lung cancer patients have neutrophils with a dysregulated NETosis program. Specifically, LCN are easier to activate, but produce fewer NETs per cell. While NETs from healthy donors and cancer patients retain tumoricidal activity, only NETs from healthy donors block cancer cell migration. Together, these data suggest that both quantitative and qualitative changes in NETs may derail neutrophil-mediated control of tumor function in lung cancer. Identifying the mechanisms for defective NETs’ formation and function may identify future therapeutic targets to enhance anti-tumor immunity and limit metastasis.

## Data Availability

The original contributions presented in the study are included in the article/supplementary material. Further inquiries can be directed to the corresponding author.
